# Endoscopic mucosal electrodes: New directions for recording and regulating gastric myoelectric activity

**DOI:** 10.3389/fsurg.2022.1035723

**Published:** 2023-01-06

**Authors:** Xu Han, Hong Zhu

**Affiliations:** Department of Gastroenterology, The First Affiliated Hospital of Nanjing Medical University, Nanjing, China

**Keywords:** gastric myoelectric activity, mucosal electrode, electrogastrogram, gastric electrical stimulation, endoscope

## Abstract

With the gradual deepening of the study of gastric motility disorders, people increasingly realize that gastric myoelectric activity plays an important role in coordinating gastric function. This article introduces the advantages of endoscopic mucosal electrodes compared with traditional electrodes. Several different types of mucosal electrodes and how to fix the electrodes by endoscope are introduced. Endoscopic mucosal electrodes can record and regulate gastric myoelectric activity, which has great value in the study of gastric motility. Endoscopic mucosal electrode technique refers to the fixation of the electrode in the designated part of the gastric mucosa by endoscope. Through endoscopic mucosal electrodes, on the one hand, we can record gastric myoelectric activity, on the other hand, we can carry out gastric electrical stimulation to interfere with gastric rhythm. Endoscopic mucosal electrodes have higher accuracy than traditional cutaneous electrodes, less trauma and lower cost than serosal electrodes. Endoscopic mucosal electrodes have a good application prospect for diseases such as gastroparesis and obesity.

## Introduction

Gastric myoelectric activity (GMA) affects gastric movement and function (1). Abnormal GMA is associated with a variety of diseases (2–5). In humans, the normal frequency of GMA is about 3 cycles per minute (3 cpm). Gastric dysrhythmias occur when interstitial cells of Cajal (ICCs) are exhausted and 3 cpm GMA disappears. Gastric dysrhythmias seem to be closely associated with nausea in many cases, as it is found in most patients with motion sickness, gastroparesis, pregnancy nausea, functional dyspepsia, and chronic nausea of unknown causes (6). Many drugs, such as morphine and glucagon, can cause gastric dysrhythmias (7). Gastric dysrhythmias induced by hyperglycemia is an important factor leading to dyspepsia in patients with diabetic gastroparesis (8). In addition, when patients have gastric dysrhythmias, the contraction of gastric body and antrum will be uncoordinated, and patients are prone to dyspepsia such as early satiety. This mechanism can be applied to limit food intake of obese patients (9). Therefore, correct recording and intervention of GMA has important clinical significance for the diagnosis and treatment of many diseases.

The traditional electrogastrogram was welcomed by many researchers because of its non-invasive nature (10). However, the accuracy of electrogastrogram is easily interfered by external factors, and abnormal GMA can not be intervened through cutaneous electrodes (11). Accurate GMA data can be obtained by temporarily placing electrodes in the serosa layer of the stomach, and in recent years, many studies have shown that serosa gastric electrical stimulation (GES) could improve gastric motility (1, 12). However, the serosal electrode is very traumatic and expensive, so it is not a perfect choice for patients (11). The purpose of this review is to introduce some research progress of endoscopic mucosal electrodes. By fixing the electrode on the gastric mucosa with endoscopic technique, not only GMA can be recorded, we can also observe spatial propagation of the gastric electrical slow waves, and visually observe whether there are diseases in the stomach, such as pyloric obstruction (10). Higher accuracy and lower trauma are the advantages of mucosal electrodes (11). In addition, the mucosal electrodes can also be used for GES, and its effect is similar to that of the serosal electrode, but the trauma and cost are much less than that of the serosal electrodes (13). Some studies have applied endoscopic mucosal electrode technique to gastroparesis (14–17), obesity (9, 18, 19), drug refractory vomiting (20) and other diseases, and achieved good results. This review introduces gastric myoelectric activity, traditional serosal electrodes and cutaneous electrodes, endoscopic mucosal electrodes, mucosal electrogastrogram, mucosal gastric electrical stimulation and so on.

## Gastric myoelectrical activity

Normal gastric myoelectrical activity (GMA) includes slow waves (pacesetter potentials or electrical control activity) and spikes (fast waves, action potentials or electrical response activity, more commonly associated with intestinal activities than the stomach) (12). The generation and propagation of the slow waves are related to interstitial cells of Cajal (ICC) (10). The normal slow waves propagate from the greater curvature of the stomach toward the pylorus with increasing amplitude and velocity, which frequency is 2.4–3.7 cycles per minute (cpm) (1). Spikes only appear after the slow waves and superimpose on it (12). Slow waves control the frequency and direction of gastric contractions, whereas the number and level of spikes determine the intensity of gastric contractions (1). Normal GMA can be perturbed by disease or surgery, generating gastric dysrhythmias and uncoupling (16). Abnormalities in GMA are classified into bradygatria (<2.4 cpm), tachygastria (>3.7 cpm) and arrhythmia (1, 11). Abnormalities in GMA may lead to gastric motility disorders, including abdominal pain, nausea, vomiting and early satiety (5, 12, 13). GMA only represent one component of control of gastric motility. For example, when the chyme enters into the duodenum, hormones such as secretin and cholecystokinin released by the “S” cells and type I secretory cells in the mucosa of the small intestine are also involved in the regulation of gastric motility (21). In addition, a complex coordination of smooth muscle contraction and innervation by the central nervous system and the enteric nervous system also regulate gastric motility (22).

## Traditional serosal electrodes and cutaneous electrodes

We know that GMA is an important factor in regulating gastric motility, then how can we record and further study it? The answer is electrodes, using electrodes to connect the stomach and catch the electrical signals we want. In clinical practice, the most accurate method is to implant the electrodes into the gastric serosa layer by operation (1, 10, 12). By using serosal electrodes, Angeli et al. (23) accurately calculated the ICCs density of chronic unexplained nausea and vomiting (CUNV) patients and plotted the abnormal gastric slow waves of CUNV patients. They successfully defined the cellular and bioelectric abnormalities of CUNV, proved that the pathophysiological characteristics of CUNV were similar to gastroparesis, which provided a new insight into the pathogenesis of CUNV. The same serosal high-resolution mapping was performed in 12 patients with diabetic or idiopathic gastroparesis, which quantified and classified the spatio-temporal mechanism of gastric slow waves in patients with gastroparesis, and found that irregular initiation, abnormal conduction and low amplitude activity may be involved to the pathogenesis of gastroparesis (24). The data recorded by serosal electrodes is comprehensive and accurate, what it highlights is the detailed mechanisms of spatiotemporal abnormalities of gastric slow waves in CUNV and gastroparesis (11). However, if this method is adopted, then patients will have to bear great pain and risk, as well as high costs (4). Therefore, the involvement of surgical procedures limits serosal electrodes' scalability.

In 1922, Alvarez et al. (25). used abdominal cutaneous electrodes to record GMA, which was called electrogastrography (EGG). EGG has been a hot research topic in recent years because it is non-invasive, easy to operate and less painful during examination. At first, the traditional EGG recording method was: a single electrode pair was fixed to the skin surface of the subject's upper abdomen, and computerized spectroscopy was used to record and compare changes in gastric slow wave frequency and power before and after stimulation with a low-fat meal (25).

Recently, recording from multiple channels from the abdomen, has achieved FDA-approval and clinical utilities. The main method was (26): four silver chloride electrodes were fixed on the skin surface of the subject's abdomen. Three electrodes were placed in the left epigastric and epigastric region, including the first electrode located at the left inferior costal margin of the midclavicular line, the third electrode located equidistant between the umbilicus and xiphoid process, and the second electrode along the midline of the first and third electrodes. The fourth electrode was placed on the right upper abdomen as a reference electrode, on the line formed by the other three electrodes. The electrodes were connected to a rectilinear recorder (R611; SensorMedics, Anaheim, CA, U.S.A.) through direct nystagmus couplers (Model 9859; SensorMedics). By using this method, Koch et al. (26). successfully recorded the EGG of 24 patients with dyskinesia-like functional dyspepsia and 24 control subjects before and after water intake with good repeatability. Some recent studies have shown that multi-channel cutaneous EGG is effective in diagnosing subsets of abnormalities and then lead to more specific therapeutic choices with very high degrees of cure as well as control of disease. About 25% of gastroparesis (GP) patients have normal amount of ICCs in their stomach because they have normal 3 cpm GMA (27). This group of patients is a unique subset of GP: obstructive GP (28). Noar et al. (27). had used cutaneous EGG with water load satiety test proved that pyloric balloon dilation improved symptoms and gastric emptying long term in obstructive GP. Wellington et al. (29). used a similar approach to show that pyloric dysfunction is a key factor in the development of functional obstructive GP and to develop a rational method for screening patients who would benefit most from surgery. The mechanism of Idiopathic rapid gastric emptying causing nausea was unclear. GMA was recorded by using multi-channel cutaneous EGG, Wang et al. (30). found that Idiopathic rapid gastric emptying and post-surgical rapid gastric emptying had similar gastric rhythm disorders, which may contribute to rapid gastric emptying.

Using the correct recording method and proper electrode arrangement, EGG can accurately record gastric slow waves (31). EGG recording performed simultaneously with serosal or mucosal electrodes had demonstrated that the dominant frequency of EGG accurately represents the frequency of gastric slow waves (32). In a study of 20 subjects, GMA was successfully recorded in 16 subjects using both cutaneous and mucosal electrodes. The results showed that the signals recorded by the mucosal electrodes and the cutaneous electrodes had the same frequency and similar waveform, and both signals changed when non-rhythmic stomach events occurred (33). A recent study showed that EGG could be as valuable as a mucosal electrode in diagnosing delayed gastric emptying. EGG was less invasive and could also act like mucosal electrodes to screen out patients who need GES (34). Some results of meta-analysis showed that in patients with nausea and vomiting syndromes (35), functional dyspepsia (36), gastroesophageal reflux disease (37), the gastric slow-wave activity recorded by EGG showed consistent abnormalities separately. However, the associated spatial patterns and symptom correlations are not represented by EGG.

EGG recording results usually require computers to remove interference from breathing, intestinal peristalsis, etc (34). In contrast, the mucosal electrodes are closer to the muscle propria of the stomach, which is the source of electrical activity in the stomach, so they are less disturbed (38). The electrode position of the EGG usually needs to cover the antrum, but the human body is highly diverse, and even a small deviation of the electrode position from the stomach can lead to a large data bias (11). However, this problem has been improved. With the guidance of CT/MRI, we can accurately find the corresponding anatomical plane and ensure the accuracy of the placement of cutaneous electrodes (39). Another approach is to locate key anatomical markers of the body, such as the xiphoid process and umbilical button, from a large number of subjects, and then use regression models to predict the position of the stomach based on these measurements (40). EGG is a kind of cumulative sine waves, the signal analysis of EGG is a technique that aggregates all gastric slow waves. Cutaneous electrodes cannot detect local slow wave activity in specific parts of the stomach (41). EGG in the detection of isolated dysrhythmias, quantitative measuring power of regional differences, and local coupling and propagation characteristics of ability is limited. In contrast, by using mucosal electrodes, a research has successfully characterized regional differences in power under normal conditions and differential dysrhythmic effects of two stimuli (glucagon and acute hyperglycaemia) (2). Although there are some limitations in the clinical value of EGG, it still plays an irreplaceable positive role in screening and diagnosing gastric motility disorders, especially its low invasiveness and ease of operation.

It is worth mentioning that in order to overcome the defects of traditional EGG, the high-resolution EGG (HR-EGG) technology has been proposed. This method consists of a dense array of cutaneous electrodes and subsequent signal processing methods, which can effectively reduce artifacts and estimate the spatial orientation of gastric slow waves (42). A recent study showed that gastric slow wave spatial patterns recorded by HR-EGG could distinguish between children with chronic nausea and healthy subjects (43). However, HR-EGG requires the accurate location of the gastric antrum under the guidance of CT and the electrode array facing the duodenum (44). The high cost and radiation of CT make this method imperfect.

## Endoscopic mucosal electrodes

To further investigate GMA, we need to find a technique that is both accurate and less invasive. Mucosal electrodes exhibit the potential for acquiring GMA from different regions of the stomach and are able to record GMA with less invasiveness than serosal electrodes (10). The biggest problem is that mucosal electrodes are easily dislodged and cannot be directed to specific mucosal locations (33). This problem has been improved through endoscopic technology and the design of new electrodes.

**Figure 1:**
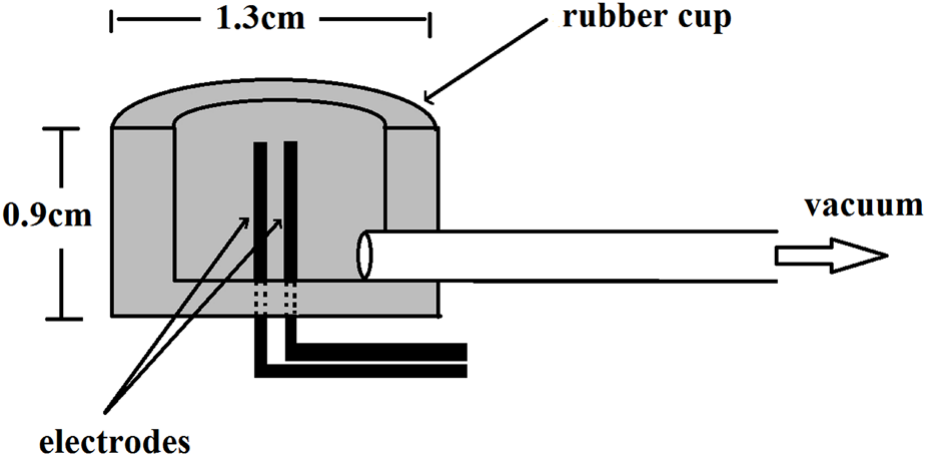
Suction-cup design mucosal electrode.

In 1970, Monges et al. (45). designed the suction-cup mucosal electrodes (shown in Figure 1). They used a probe which had two rubber cups 3 cm apart in the tip to insert transorally into the stomach. Two stainless steel electrodes, 2 mm apart and 0.6 mm in diameter, were positioned inside each rubber cup. The cavity of the rubber cup was connected to a polyethylene tube through a metal tube. When the probe reached the designated part of the stomach, a negative pressure was given to the cavity through the polyethylene tube so that the electrodes could be attached to the gastric mucosa tightly. The Ag-AgCl electrodes used by Hamilton et al. (33). were also adopted a similar design.

**Figure 2:**
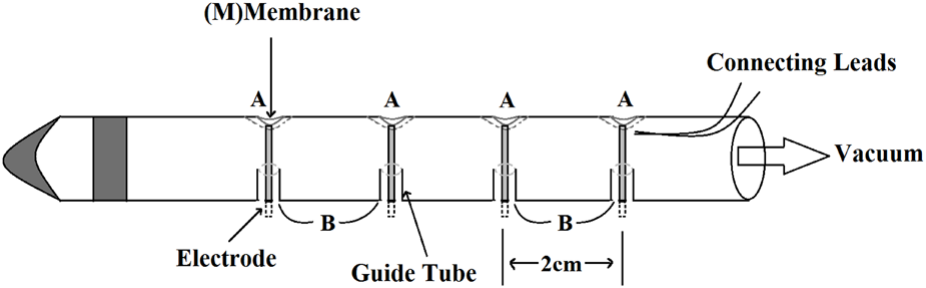
Schematic diagram of the mucosal electrode.

However, the negative pressure of this suction-cup electrodes will pull the mucosa away from the underlying smooth muscle tissue (the source of the electrical signals), thus degrading the recorded signals (46). To reduce the error, Familoni et al. (46). used a similar principle to design a new electrode (shown in Figure 2). A and B round holes were cut out in a tube, then Ag-AgCl electrodes were bonded on rubber membranes (M), which covered the holes A. When a negative pressure was given to the tube, the rubber membranes would deform and push the electrodes out of the holes B, so that the electrodes could be attached to the gastric mucosa tightly. This type of electrodes can be mounted in the Levine type 900–51 gastric tube [manufactured by Cutter (Canada) Ltd.] and record GMA in any part of the stomach.

**Figure 3:**
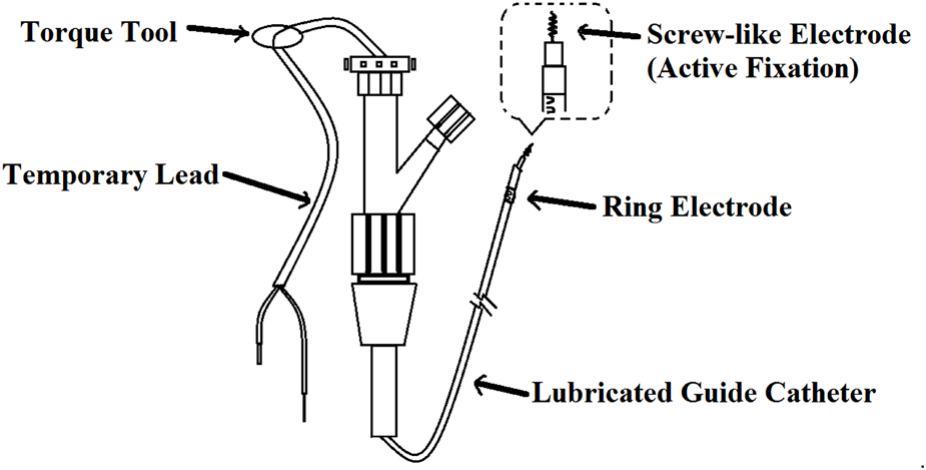
Temporary intravenous cardiac pacing lead. The screw-like electrode was screwed into the mucosa by rotating the Guide Catheter. The ring electrode was attached to the mucosa by a clip.

Nowadays, with the help of endoscopic technology, the temporary cardiac pacing lead (47), the fetal scalp monitor lead (48) and other stainless steel lead (48) constructed with corkscrew tine can be easily attached to the gastric mucosa. The method (14) is as follows (shown in Figure 3): a temporary lead was inserted into the gastric mucosa at the junction of the gastric antrum and body through a standard 140 cm endoscope and a 7F auxiliary channel. The temporary lead had an internal bipolar electrode pacemaker lead, which were composed of a screw-like electrode (active fixation) and a ring electrode. The lead was inserted into the stomach through the nasal cavity under pharyngeal anesthesia. Under the guidance of endoscope, the screw-like electrodes were inserted clockwise into the gastric mucosa to a depth of about 2–3 mm. Then 3–5 titanium clips were uesd to attach the ring electrode to the mucosa.

It is difficult to accurately evaluate the GMA with a single electrode (11). Angeli et al. (10) developed a new mucosal electrode array to quantify the propagation and morphological characteristics of GMA. The electrode array consisted of 64 electrodes distributed on 8 strands. Each strand encompassed 8 electrodes with 7 mm inter-electrode spacing. There was an airbag in the middle. When the airbag was not inflated, the electrode array could be sent into the stomach through endoscope. The air bag was then inflated to keep the electrode in close contact with the surface of the gastric mucosa during recording. An experiment was carried out on 14 piglets, and the abnormal GMA was successfully located and classified from the surface of gastric mucosa, which was consistent with the known data in the pig model.

## Applications of endoscopic mucosal electrodes

### Mucosal electrogastrogram

With the help of endoscope, we can easily fix the above electrodes to the gastric mucosa. Then we only need to connect the external lead to the specific instrument to get the mucosal electrogastrogram. At first, the electric signals were AC coupled to a Beckmann polygraphinclude such as Beckman R511 (33) and Beckman R611 (46). Now with the development of electrogastrography technology, the recording of GMA has become more convenient and accurate, such as the EGG recorder (Sandhill Scientific, Highlands Ranch, Colorado) (47) and the multi-channel electrogastrograph (POLYGRAMNETTM, Medtronic Functional Diagnostics A/S, Denmark) (18).

Although there are few studies on mucosal electrogastrogram, some studies have shown that the abnormality of mucosal electrogastrogram is related to some diseases. In 2004, Coleski et al. (3). recruited 12 healthy volunteers, including 7 men and 5 women, aged 19–43 years. With the help of a gastroscope, the mucosal electrodes were fixed 12, 7, and 2 centimeters away from the pylorus, along the greater and lesser curvature of the stomach. Each volunteer underwent endoscopic mucosal recording studies under two separate test conditions (control and intravenous glucagon) on 2 days separated by ≥3 days in random order. In control group, mucosal electrogastrogram clearly showed the slow wave energy gradient from the proximal to distal stomach. In intravenous glucagon group, mucosal electrogastrogram showed that glucagon disrupted normal slow waves and finally turned it into a slow, bradygastric rhythm. In 2009, Coleski et al. (2). used a similar method to record mucosal electrogastrogram of healthy volunteers in normal and hyperglycemic (250 mg/dl) states. The results of mucosal electrogastrogram showed that hyperglycaemia could cause uncoupling of normal slow wave and isolated tachygastrias. The above studies suggest that there are differences in mucosal electrogastrogram between normal people and patients with hyperglycaemia. Some studies have confirmed that up to 70% of patients with diabetic gastropathy have tachygastria and bradygastria, which are associated with abdominal pain, nausea, vomiting and early satiety (49). We can accurately detect GMA in these patients through mucosal electrgastrogram, thus guiding the treatment to improve their upper gastrointestinal symptoms (50).

Gastroparesis is a complex disorder of gastric motility, and abnormal GMA is often reflected in the mucosal electrogastrogram (36). Hamilton et al. (33). used mucosal suction electrodes to record the GMA in 20 volunteers, and demonstrated that the amplitude of the GMA increased with gastric contractions. They also explored the changes of mucosal electrogastrogram during nausea in 4 patients with gastroparesis. The results showed that the patient's GMA was approximately 3 times per minute under normal conditions, but tachygastria was revealed on mucosal electrogastrogram when nausea appeared. This condition was also seen in patients with atrophic gastritis and delayed gastric emptying. In one study (5), 111 patients with refractory gastroparesis who received gastric electrical stimulation were included. Two mucosal electrodes were placed simultaneously through endoscope in 69 patients and single mucosal electrode was placed in other 42 patients. The average frequency and amplitude were recorded at each electrode. Compared with the single-point electrode, the two low-resolution electrodes could record gastric electrical transmission in more detail. The analysis of multi-electrode mucosal electrogastrograms is a useful method to understand the pathophysiology of gastroparesis (5). Although gastroelectric dysrhythmia often occurs in gastroparesis, the reliability of electrical diagnosis has always been controversial. Recently, high-resolution mapping has appeared, which brings hope for the clinical application of electrical diagnosis (11). However, at present, serosal electrodes are used in high-resolution mapping, which limits its clinical application due to its huge trauma. Multi-electrode mucosal electrogastrogram is considered to be a bridging technique until a new high-resolution mapping technique is developed (10).

### Mucosal gastric electrical stimulation

**Figure 4:**
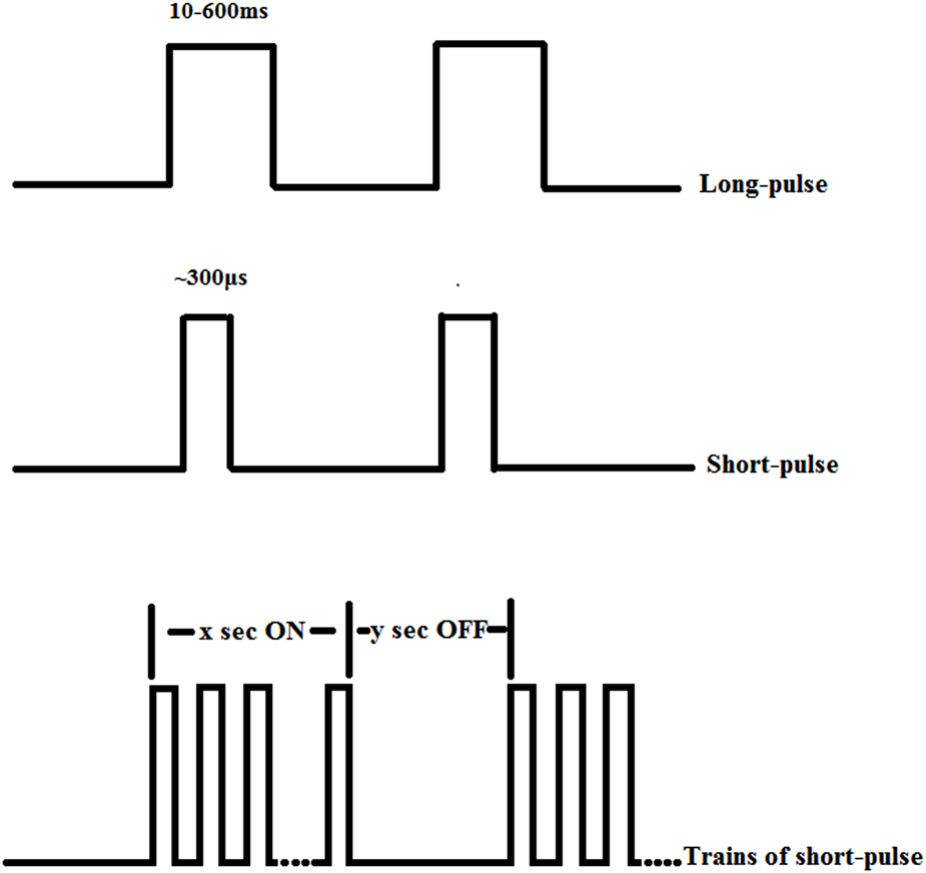
Three different methods of gastric electrical stimulation.

Endoscopic mucosal electrodes not only enable assessment of GMA, but also provide interventions for abnormal GMA, such as gastric electrical stimulation (GES). In 1963, Bilgutay et al. (51) firstly used GES in a dog model, and they found that gastric contraction and gastric emptying increased in dogs. Since then, GES has been recognized as a potential treatment for gastric motor dysfunction. In 1990 s, there were two main ways of GES: one using higher energy with close to the physiological frequency, and another using lower energy with several times higher than physiological frequency. Most clinical activities have utilized higher frequency and lower energy (52). GES consists of a series of pulses, usually rectangular, with a constant current or voltage, involving several electrical stimulation parameters, including frequency, pulse width and amplitude, usually in a few milliamperes (1, 12). As shown in Figure 4, GES is divided into long-pulse stimulation, short-pulse stimulation and stimulation with trains of short-pulses (1).

According to the number of electrodes, it can be divided into single channel GES and multi-channel GES (12). Long-pulse stimulation is the most widely used and can pace or carry natural gastric slow waves with a pulse width of 10–600 ms (1, 12). It's stimulation frequency is usually near the physiological frequency of gastric slow waves (12). Compared with long- pulse stimulation, the pulse width of short-pulse stimulation is shorter, about a few hundred microseconds, and the stimulation frequency is usually several times higher than the physiological frequency of gastric slow waves (1). Because the long-time constant of smooth muscle is about 100–300 ms, only long-pulse stimulation can change gastric slow waves, while short-pulse or stimulation with trains of short-pulses has little effect on gastric slow waves (53). However, if the stimulation frequency is high enough and the subsequent pulse is close enough to the previous pulse, due to the slow voltage attenuation at the end of the pulse, two or more short pulses can be regarded as a long pulse, thus changing the gastric slow waves (12). The stimulation with trains of short-pulses consists of a repetitive short pulse sequence and contains the following two signals: (1) 5–100 Hz high frequency continuous short pulses (2) the control signal of the switching pulse, such as x second “on” and y second “off”, x and y add to determine the frequency of the pulse sequence (1, 12). In most previous studies, gastric electrical pacing used a pair of electrodes to stimulate the area near proximal gastric antrum, that is, forward single-channel gastric pacing, which can be used to treat gastroparesis patients (16). But in some studies, by contrast, electrodes were placed close to the pylorus to reverse the direction of stomach contraction, called retrograde pacing, which could be used to reduce food intake in obese patients (18, 54).

Although there are few clinical cases of GES through mucosal electrodes, there is a trend of increasing (shown in Table 1).

**Table 1:** Clinical applications of mucosal GES in recent years.

Time	Model	Position of electrodes	Instrument	Conclusions
2005	Healthy humans	5 cm above the pylorus	A universal pulse generator (Acupulser, model A310, World Precision Instrument, Inc., Sarasota, FL, USA): Frequency 9 cpm; pulse width 500 ms;amplitude 5 mA	Acute RGES can result in a series of symptoms associated with dyspepsia, which is beneficial to the treatment of obesity. Optimal parameter should be determined according to the individual sensitivity to electrical stimulation.
2005	Patients with gastroparesis	The junction of the antrum and the body of the stomach	Enterra: Frequency14 Hz; amplitude5–10 mA; pulse width 330 μs; cycle ON 0.1–1.0 s; cycle OFF 5.0–4.0 s.	Endoscopic placement of mucosal GES electrodes are safe and effective in patients with gastroparesis.
2005	Healthy humans	The greater curvature 5 cm above the pylorus	A universal pulse generator (Acupulser, model A310, World Precision Instrument, Inc., Sarasota, FL, USA): Long-pulse:frequency 9 cpm; pulse width 500 ms; amplitude 5 mA, 10 mAor15 mA; Pulse train:2 s ON, 3 s OFF; frequency20 Hz; pulse width5 ms, 10 ms or 20 ms; amplitude10 mA, 15 mA or 20 mA.	GES *via* the distal stomach reduces gastric accommodation and delays gastric emptying.
2006	Healthy humans	The greater curvature 5–6 cm away from the gastroesophageal junction.	An adjustable stimulator (model A310, World Precision Instruments, Inc., Sarasota, FL): Amplitude5–10 mA; pulse width 100–500 ms; frequency 9 cpm; 10 min ON, 10 min OFF.	GES using temporary mucosal electrodes decreases food intake as well as maximum intake of water,and has a tendency of delaying gastric emptying. It may have a potential application for the treatment of obesity.
2011	A man with diabetic gastroparesis	The junction of the gastric body and antrum	Enterra	ENDOStim is the preferred method for placement of electrodes for temporary GES.
2013	Patients with gastroparesis	The junction of the gastric body and antrum	Enterra: Impedance: 200–800 Ω; current: 5 mA; cycle time: 0.1 s ON, 5 s OFF	A trial of temporary, endoscopically delivered GES may be of predictive value to select patients for laparoscopic- implantation of a permanent GES device.
2015	Patients with gastroparesis	The junction of the gastric body and antrum	Implantable pulse generator (Medtronic 3116)	GES may be an effective therapy for treating the symptoms of gastroparesis with normal gastric emptying.
2016	Patients with drug refractory, cyclic vomiting syndrome	The junction of the gastric body and antrum	Enterra: Frequency, 14 Hz; intensity, 5 mA; pulse width, 330 μs; cycle ON, 0.1 s; cycle OFF, 5 s.	In a small group of drug-refractory CVS patients, treatments with temporary and permanent GES significantly reduced the severity of gastrointestinal symptoms and frequency of hospital admissions.
2018	Patients with gastroparesis	10 cm from the pylorus at the greater curvature and separated approximately 1 cm	Enterra: Frequency, 14 Hz; intensity, 5 mA; pulse width, 330 μs; cycle ON, 0.1 s; cycle OFF, 5 s.	Laparoscopic implantation of a temporary GES system predicts the outcome of permanent GES and is cost-saving.

Mucosal GES can treat gastroparesis, relieve symptoms, and the effect is similar to that of permanent implantable gastric electrical stimulator (17). Especially when the effect of drug treatment is not good, GES is a good choice for patients with gastroparesis (13). In 2005, Ayinala et al. (14) performed GES on 20 gastroparesis patients through mucosal electrodes, 13 of whom later received permanent gastric electrical stimulators. The effects of temporary GES and permanent GES were compared by mean vomiting frequency score (VFS), total symptom score, days of symptom improvement, electrode impedance and gastric emptying test. The results showed that GES with mucosal electrode was safe and effective, and the result was consistent with that of permanent GES. In 2015, Sanjeev et al. (17) selected 452 patients with gastroparesis and divided them into normal gastric emptying group (*n* = 137), delayed gastric emptying group (*n* = 273) and rapid gastric emptying group (*n* = 42) by radionuclide gastric scan. The main symptoms of all patients were nausea, followed by early satiety, followed by abdominal pain and distension, and finally vomiting. GES was performed through endoscopic mucosal electrodes in 379 patients. After treatment, gastric retention time in delayed group was decreased, while gastric retention time in normal group and rapid group was increased. At the same time, the symptoms of vomiting and nausea were improved in all three subgroups. In 2016, Grover et al. (20) conducted an one-year non-randomized clinical study. After 11 patients with intractable periodic vomiting syndrome received mucosal GES and permanent GES, the total symptom score decreased by 68% and 40%, respectively, and the number of hospitalization events decreased from 9.14 (±7.21) to 1.5 (±1.00) per year.

Permanent implantable gastric electrical stimulator can treat gastroparesis, but it is not suitable for all patients. If the operation is carried out blindly, it will bring great harm to the patients. According to the response of patients to temporary mucosal GES, the curative effect of permanent GES can be predicted. In 2013, Sarela et al. (16) performed mucosal GES in 51 patients with gastroparesis, of whom 39 were treated with good results and were selected for permanent GES treatment. 31 patients were followed up for 10 months, of which 22 responded well to permanent GES. In 2018, Florian et al. (13) performed temporary GES on six gastroparesis patients who had ruled out gastric outlet obstruction by endoscopy. Of the 6 patients, 4 had an effect on temporary GES. Of the 4 effective patients, 3 received permanent gastric pacemaker implantation. One invalid was implanted with a permanent gastric pacemaker in another institution. During the 9-month follow-up, the gastroparesis cardinal symptom index of patients who had an effect on temporary GES remained low, while the index of ineffective patients increased. The results of this study suggest that temporary GES through mucosal electrodes can predict the efficacy of permanent gastric pacemakers, thereby reducing cost and risk.

Mucosal GES could also be used to reduce food intake in obese patients. In 2005, Yao et al. (18) temporarily placed bipolar mucosal electrodes 5 cm above the gastric pylorus of 12 healthy volunteers, followed by acute retrograde gastric electrical stimulation (RGES). The common symptoms during stimulation were satiety, abdominal distension, discomfort, pain, tingling, nausea and so on. When the amplitude or pulse width was adjusted to a higher scale, the total symptom score of each subject increased significantly. In another article in the same year, Yao et al. (19) said that GES of the distal stomach through mucosal electrodes could delay gastric emptying with little side effects. These inhibitory effects were related to the visceral sensitivity of individuals to gastric stimulation. In 2006, Liu et al. (9) performed GES on 12 healthy volunteers for 3 days through mucosal electrodes. Compared with the control group (1093 ± 417 ml), the maximum water uptake of GES group (894 ± 326 ml) decreased. Food intake was also reduced in the GES group (paired 0.012). Compared with the control group, gastric emptying in the GES group was delayed 45 min after the meal, but there was no significant change after the meal. These results suggested that the use of endoscopic mucosal electrodes for GES might provide hope for the treatment of obese patients.

Mechanisms that may explain the beneficial effects of GES are still under investigation. Many studies have found that GES does improve the symptoms of patients with intractable symptomatic gastroparesis over a long period of time, but the exact mechanism remains unclear (53). A recent study shown that GES could increase vagal efferent activity and thalamic metabolic activity. The enhanced efferent autonomic function of the vagus nerve and the reduced sensitivity of the stomach to volume expansion may be the mechanisms by which GES may improve nausea and vomiting symptoms (55). However, in previous studies, GES did not significantly increase the level of glucose metabolism in the brain of patients with gastroparesis (55). Changes in GMA can induce changes in gastric hormone levels, such as gastrin and ghrelin levels (56). Brain—stomach coupling plays an important role (57). This may be an important mechanism for GES to change the food intake of patients, but further research is needed.

In short, mucosal GES can be performed through endoscopic mucosal electrodes, which has the potential to treat gastroparesis, obesity and drug-refractory vomiting. The efficacy of permanently implantable gastric electrical stimulators can also be evaluated. Under pharyngeal anesthesia, the leads to the electrodes are drawn out of the nasal cavity and retained. The mucosal electrodes can be retained in the stomach for 2–3 days, during which time we can record GAM multiple times or continuously by external leads and perform GES. At the same time, combined with clinical symptom rating scale, Water Load Test, Scintigraphy and other methods, we can assess gastric activity more comprehensively (9, 14). Based on the results of the gastric motility assessment, we can adjust the energy, frequency, number of GES at any time, and decide whether to perform permanent GES surgery. However, most of the current mucosal electrodes are temporary and fixed in the stomach for no more than 48–72 h (48). In addition, due to the softness, elasticity and easy movement of the gastric mucosa along the muscle surface, it is easy to inhibit the electrical signal of the mucosal electrodes to penetrate into the muscle layer, resulting in the failure of GES (48).

## Conclusions

Endoscopic mucosal electrodes can not only record GMA, but also interfere with gastric function through GES, which is of great value in the study of gastric electrophysiology and the treatment of gastric motility disorders. The main advantages of endoscopic mucosal electrodes are high accuracy, less trauma, and the integration of diagnosis and treatment. Although there is no clear standard for gastroelectric diagnosis at present, and more advanced mucosal electrodes need to be developed to overcome the problems of poor contact and low resolution, the combination of endoscopic technology and mucosal electrodes must be of great clinical value.
